# A dual-band high-gain beam steering antenna array for 5G sub-6 GHz base station

**DOI:** 10.1038/s41598-024-75822-2

**Published:** 2024-11-03

**Authors:** Salman Ilahi Siddiqui, Shahid Bashir, Awais Khan, Salman Ghafoor, Imran Aziz

**Affiliations:** 1 Department of Electrical Engineering, University of Engineering and Technology (UET), Peshawar, Pakistan; 2 Department of Electrical Engineering, University of Science and Technology (UST), Bannu, Pakistan; 3 School of Electrical Engineering and Computer Science, National University of Sciences and Technology (NUST), Islamabad, Pakistan; 4 Department of Electrical Engineering, Mirpur University of Science and Technology, Mirpur (AJK), Pakistan; 5 Department of Physics and Astronomy, Uppsala University, 75120 Uppsala, Sweden

**Keywords:** Base station array, Beam steering, Gain enhancement, Dual-band antenna, Engineering, Electrical and electronic engineering

## Abstract

An antenna array having a size of 45 $\:\times\:$ 40 cm^2^ (5.7 $\:\times\:$ 5 $\:{\lambda\:}_{0}$^2^) and consisting of four pairs of printed U-shaped dipoles positioned above a metal reflector, for 5G Sub-6 GHz base station applications, is designed and tested. The array consists of eight excitation ports, one port for each dipole. Four parasitic square patches are etched on the bottom side of the dipole arms for producing radiations in 2.2 GHz and 3.8 GHz bands. The size of the reflector and height of the dipoles are optimized in order to enhance antenna gain up to 11.5 dB at 2.2 GHz and 14.5 dB at 3.8 GHz. Beam steering up to 20$\:^\circ\:$ is achieved, using phase shifted simultaneous excitation of different ports. The proposed antenna array not only fulfills 5G base station requirements but is also simple and compact as it only requires eight ports to achieve dual-band, high-gain and beam steering operation in a single design. It also offers a unique feature of dual-sector coverage per panel, which results in an increased coverage capacity of the base station without increasing the system resources.

## Introduction

With the advent of the fifth generation (5G) technology, cellular communication has entered a new era of connectivity that promises very high speeds, extremely low latency, and large network capacity^1^. It is a standardized and enhanced wireless communication interface^2^. The 5G system incorporates an expanded capacity to facilitate advanced user experiences, support innovative deployment models, and provide new services^3^. There are two bands that have been dedicated for the deployment of 5G; sub-6 GHz and mmWave^4^. The sub-6 GHz band includes frequencies below 6 GHz whereas the mmWave band includes frequencies ranging from 24 GHz to 80 GHz^5^. The sub-6 GHz band offers a balance between coverage and capacity, making it suitable for 5G mobile communication^6^. One of the most important component of wireless network coverage in a cellular system is the base station antenna. A base station antenna is used for the transmission and reception of wireless signals to and from the mobile devices within their coverage area, also known as the sector^7^. Base station antennas used for cellular communication systems, are of two basic types; omni-directional antennas and directional antennas. The omni-directional antennas offer low capacity and extended coverage and are therefore suitable for rural areas. On the other hand, directional antennas also referred to as sector antennas, offer high capacity and targeted coverage and are therefore suitable for urban areas^8^. In order to enhance base station coverage and improve signal quality, antennas achieving pattern diversity are preferred^9^. The other design challenges associated with 5G directional base station antennas include multi-band or wideband operation^10,11^, high-gain radiation pattern^12,13^, beam steering capability^13,14^ and overall size or complexity of the system^15,16^. Furthermore, it is also important to make 5G networks compatible with the existing 3G/4G standards^17^. Therefore, the base station antennas have to be multiband or wideband so as to support multiple generations of cellular technology.

In recent years, different multi-band and wideband antennas for base stations consisting of patch antennas, loop radiators and printed dipoles, have been used^18,19^. The advantage of using printed dipole antennas is that these can often be directly connected to coaxial cables or feed balun, which can effectively improve their radiation performance^20^. For dipole antennas, different shapes and arrangements like bow-tie^21^, coupled^22^, crossed^23,24^ and folded dipoles^25^ have been presented. With the advancements of cellular communication systems in the recent years, researchers have been mostly interested in radiation-reconfigurable printed antennas. The requirements for a base station antenna, include steerable antenna beam with high gain and directivity, which can be achieved by developing antenna arrays^26^. An antenna array is composed of multiple antenna elements and each element is connected to a phase shifter. The phase shifter is used to change the phase angle of the excitation signals which results in the constructive and the destructive interference, and hence not only changing the direction of the beam but also varying the beamwidth^27–29^. These techniques of beam steering and pattern reconfiguration have also been implemented using other approaches like switchable diodes^30,31^, parasitic patches^32,33^ and metasurfaces^34–36^.

Another important aspect of 5G base station antennas, is high directivity and gain. For achieving highly directed beams, different designs have been presented but at the expense of their overall size and complexity^8^. The size of the antenna array is not only expressed in terms of its physical dimensions (cm^2^) but also electrical dimensions ($\:{\lambda\:}_{0}$^2^), where $\:{\lambda\:}_{0}$ is the free space wavelength of the array, which is evaluated at the center frequency of its 5G sub-6 GHz band. In^37^, a 256 $\:\times\:$ 21.5 cm^2^ (49.5 $\:\times\:$ 4.1 $\:{\lambda\:}_{0}$^2^) array with 64 ports has been proposed to achieve a high gain of 18 dB, but is a single band antenna system which also does not have beam steering capability. In^15^, 14.8 dB gain was recorded using an array of 60 $\:\times\:$ 14 cm^2^ (4.4 $\:\times\:$ 1 $\:{\lambda\:}_{0}$^2^) with 10 ports but does not incorporate beam steering. A gain of 16.7 dB was achieved by^12^ with an array of 194.4 $\:\times\:$ 8.6 cm^2^ (24 $\:\times\:$ 1 $\:{\lambda\:}_{0}$^2^) and 48 ports but is a single band array which also does not have beam steering capability. In^38^, 96 $\:\times\:$ 12 cm^2^ (11.5 $\:\times\:$ 1.4 $\:{\lambda\:}_{0}$^2^) array with 16 ports has been proposed to achieve a gain 16.6 but does not incorporate beam steering. In^13^, a very high gain of 19.5 dB was recorded using an array of 44.5 $\:\times\:$ 29.6 cm^2^ (5.3 $\:\times\:$ 3.6 $\:{\lambda\:}_{0}$^2^) with 24 ports but does not support multiband operation. An array of 88 $\:\times\:$ 14.3 cm^2^ (6.2 $\:\times\:$ 1 $\:{\lambda\:}_{0}$^2^) array with 16 ports was presented in^11^ to achieve a high gain of 19 dB but unable to incorporate beam steering. In^14^, a metal reflector has been used for giving additional directivity and hence increasing the antenna gain up to 17.4 dB but the design does not support multiband capability. Current research on 5G base station arrays hasn’t identified a single antenna array model that addresses all these key base station challenges in a single design.

In this study, a 5G sub-6 GHz base station antenna array, is proposed and tested. The array offers dual-band, high gain, beam steering capability. It consists of four pairs of printed U-shaped dipoles positioned above a metal reflector. The overall design is compact as it has a physical size of 45 $\:\times\:$ 40 cm^2^ and electrical size of 5.7 $\:\times\:$ 5 $\:{\lambda\:}_{0}$^2^ and also simple as it only requires eight ports for excitation. The size of the reflector and the height of the dipoles are optimized in order to enhance antenna gain up to 11.5 dB at 2.2 GHz and 14.5 dB at 3.8 GHz. In addition to being compact in size, the proposed model also satisfies other requirements of 5G base station arrays in a single design. The proposed array also offers a unique feature of dual-sector coverage per panel, which means that in a base station operation, by using a single array panel, two sectors can be covered. This results in an increased coverage capacity of the base station without increasing the system resources. This type of coverage technique has not been proposed in the literature related to 5G base stations.

## Antenna design and working

### Antenna element

**Fig. 1:**
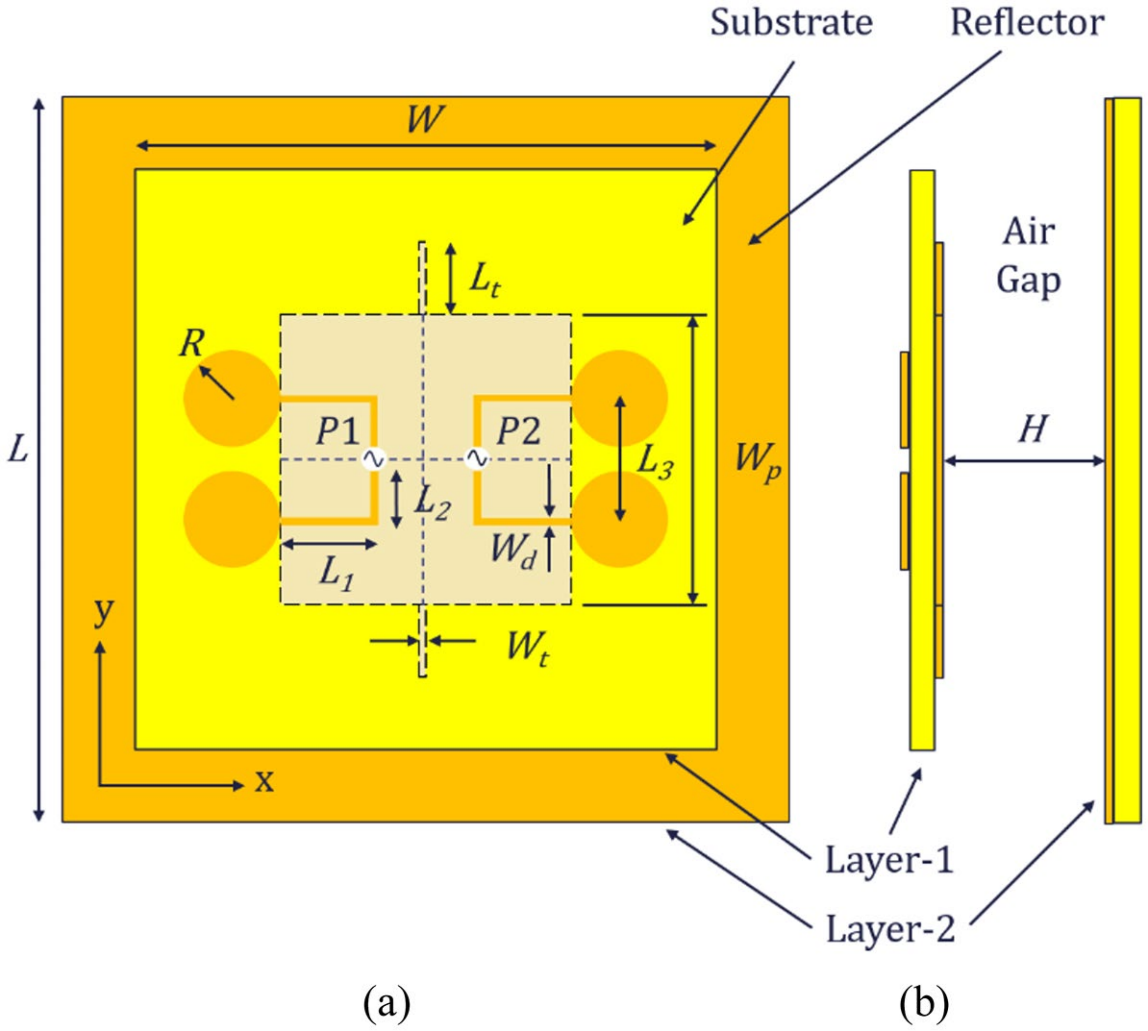
Profile of proposed antenna element. (**a**) Front view, (**b**) Side view.

The proposed antenna element, consists of two layers; layer-1 (top layer) and layer-2 (bottom layer), as shown in Fig. 1. Layer-1 consists of two center-feed U-shaped half-wave dipoles with circular radiators of radius,$\:\:R$. The dipoles are excited using two ports named as $\:P1$ and $\:P2$. Each dipole has two bent arms, each of length, $\:{L}_{1}\:+\:{L}_{2}$. The dipoles are printed on the top side of a square substrate. The center to center distance between the radiators is $\:{L}_{3}$. The substrate used here is an FR-4, with a thickness of 0.8 mm, relative permittivity of 4.3 and a side length, $\:W$. The two arms have the same width,$\:\:{W}_{d}$. A parasitic square patch with side length, $\:{W}_{p}$ is etched in the center of the bottom of layer-1 for impedance matching. Pattern diversity is achieved by using a pair of tuning stubs having length, $\:{L}_{t}$ and width, $\:{W}_{t}$ is etched along the y-axis on the same side as that of the patch. Layer-2 also consists of a square FR-4 substrate, with copper on one side, having side length, $\:L$. This layer acts as reflector for improving the antenna gain. The two layers are separated by an air gap of height, $\:H$. The length of our proposed half-wave dipole, $\:{L}_{D}$ and the corresponding wavelength, $\:{{\uplambda\:}}_{D}$ are given by expressions (1) and (2) respectively.

1 $$\:{L}_{D}=2\:\left(R+\:{L}_{1}+\:{L}_{2}\right)$$

2 $$\:{\lambda\:}_{D}=2\:\left({L}_{D}\right)$$

**Fig. 2:**
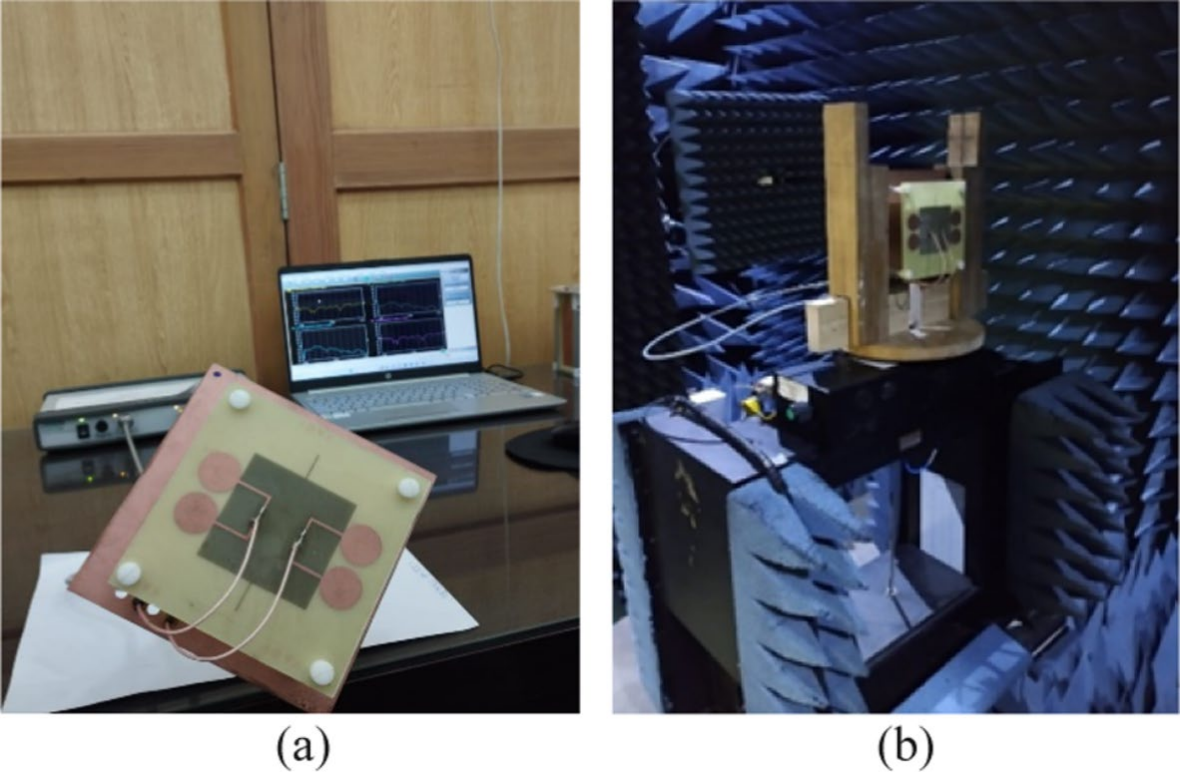
Photograph of prototype of antenna element (**a**) with network analyzer (**b**) in anechoic chamber.

The proposed model is designed and simulated in CST MW Studio and its optimal dimensions are given in Table 1. The prototype of the proposed antenna element is also fabricated as shown in Fig. 2. A 50 Ω coaxial cable is connected to the center of each dipole. The two layers are separated using plastic posts. The S-parameter measurements were taken using a Vector Network Analyzer and radiation patterns were measured in the Anechoic Chamber.

**Table 1:** Optimal dimensions of the antenna element.

Parameter	$\:L$	$\:W$	$\:{L}_{1}$	$\:{L}_{2}$	$\:{L}_{t}$	$\:{L}_{3}$
Value (mm)	150	120	18	12	15	24
Parameter	$\:H$	$\:\:R$	$\:{W}_{p}$	$\:{W}_{d}$	$\:{W}_{t}$	–
Value (mm)	34	10.5	60	1.5	1	–

**Fig. 3:**
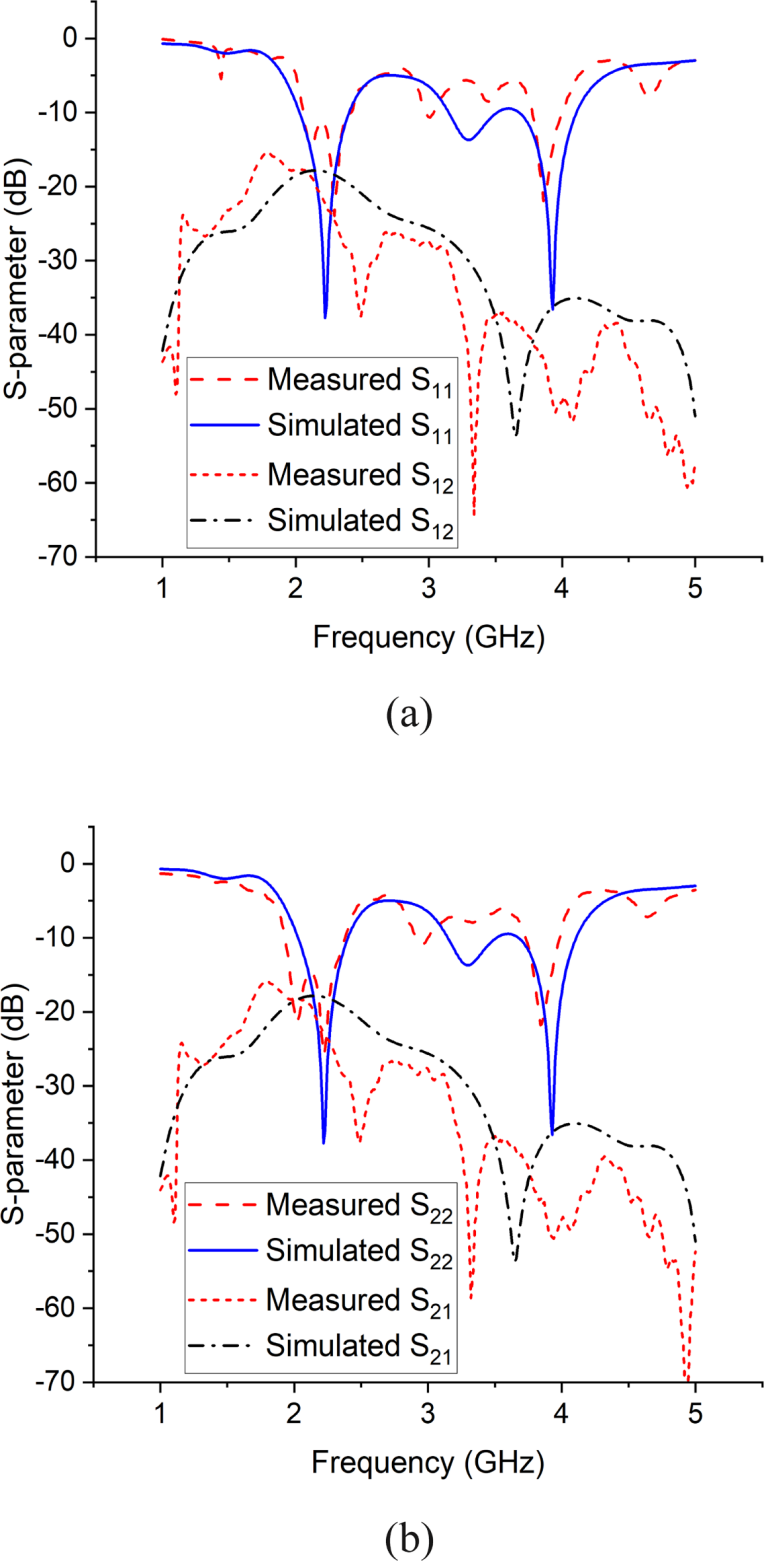
Simulated and measured S-parameters of antenna element (**a**) S_11_ and S_12_, (**b**) S_22_ and S_21_.

Figure 3 shows the simulated and measured S-parameters for port 1 and 2 of the antenna element.

**Fig. 4:**
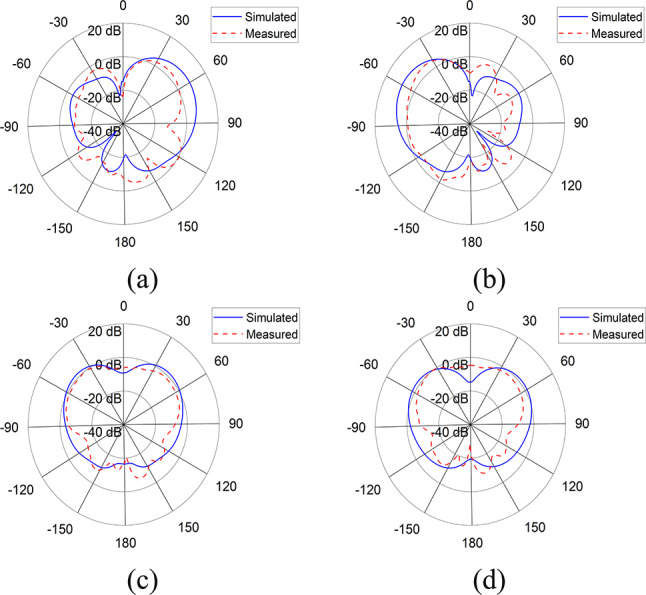
Simulated and measured radiation patterns of antenna element at 3.9 GHz in (**a**) Azimuth plane for $\:P1$, (**b**) Azimuth plane for $\:P2,$ (**c**) Elevation plane for $\:P1$, (**d**) Elevation plane for $\:P2$.

Figure 4 shows the simulated and measured radiation patterns in azimuth (xz) plane and elevation (yz) plane for port 1 and 2 of the antenna element at 3.9 GHz.

### Antenna array

**Fig. 5:**
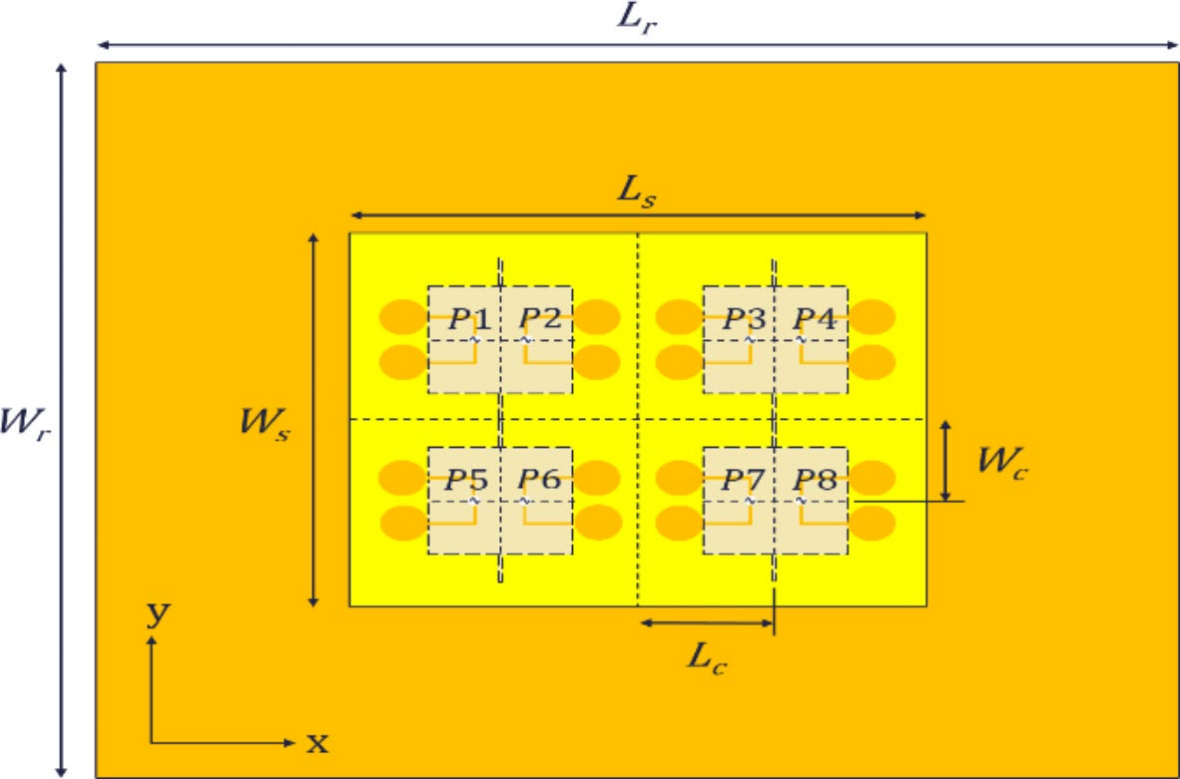
Front view profile of proposed antenna array.

In order to further increase the antenna gain and improve directionality, an antenna array is proposed, consisting of four antenna elements as shown in Fig. 5. The array also consists of two layers; layer-1 (top layer) and layer-2 (bottom layer). Layer-1 consists of four pairs of dipoles with a total of eight excitation ports named as $\:P1$ to $\:P8$, one port for each dipole. The dipoles are printed on the top side of an FR-4 substrate with a thickness of 0.8 mm, relative permittivity of 4.3 and size of $\:{L}_{s}$ $\:\times\:$ $\:{W}_{s}$. Four parasitic square patches are etched on the bottom of the same substrate. Layer-2 also consists of an FR-4 substrate with copper on one side and having a size of $\:{L}_{r}$ $\:\times\:$ $\:{W}_{r}$. As, this copper reflector is acting as a perfect electric conductor (PEC), so keeping in view the two resonant frequencies of the antenna model, $\:{f}_{1}$ and $\:{f}_{2}$, the value of the air gap, for the antenna array is re-evaluated as $\:{H}_{R}$ from expression (3), in which $\:{\lambda\:}_{R}=\:c/{f}_{3}$ where, $\:c\:$is the speed of light and $\:\:{f}_{3}=\:\left({f}_{1}+\:{f}_{2}\right)/2$.

3 $$\:{H}_{R}=\:\frac{{\lambda\:}_{R}}{4}$$

**Fig. 6:**
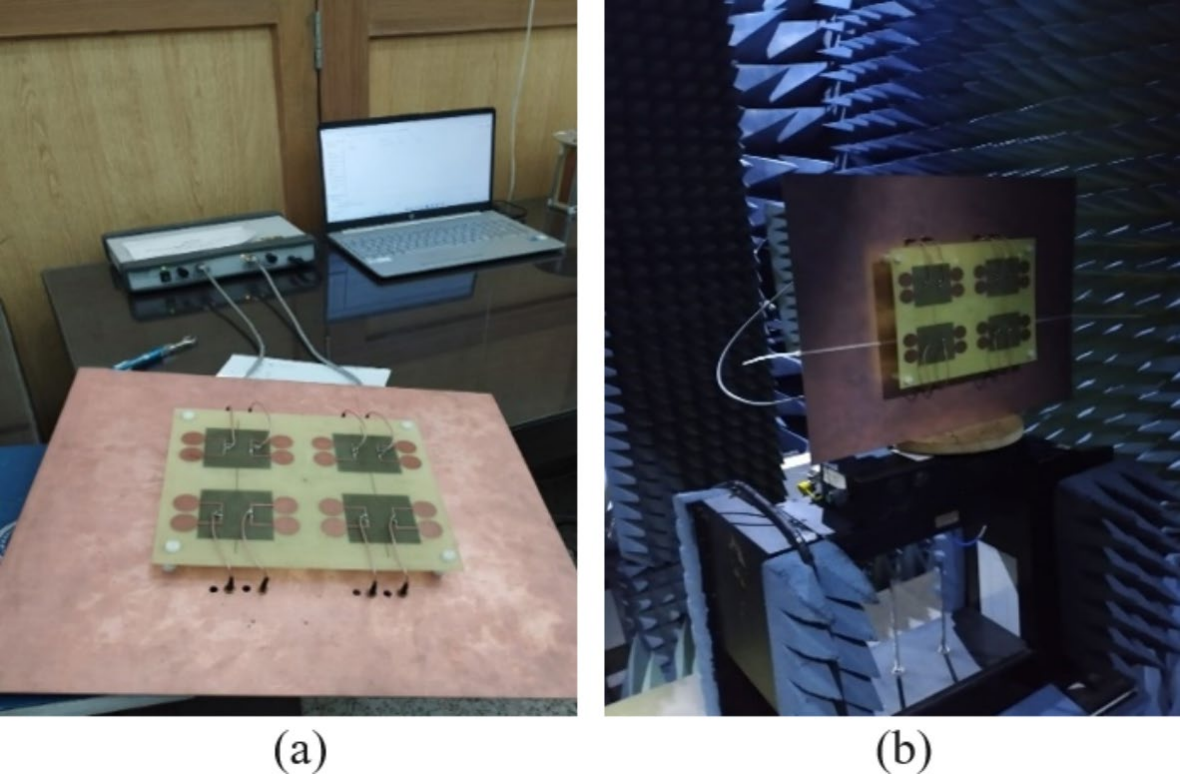
Photograph of prototype of antenna array (**a**) with Network Analyzer, (**b**) in Anechoic Chamber.

The proposed model is designed and simulated in CST MW Studio and its optimal dimensions are given in Table 2. The other dimensions like dipole radius, arm length and width, patch size, stub size are similar to that of the antenna element. The prototype of the proposed array was also fabricated as shown in Fig. 6.

**Table 2:** Optimal dimensions of the antenna array.

Parameter	$\:{L}_{r}$	$\:{L}_{s}$	$\:{L}_{c}$	$\:H$
Value (mm)	450	240	60	25

Parameter	$\:{W}_{r}$	$\:{W}_{s}$	$\:{W}_{c}$	–
Value (mm)	400	210	45	–

**Fig. 7:**
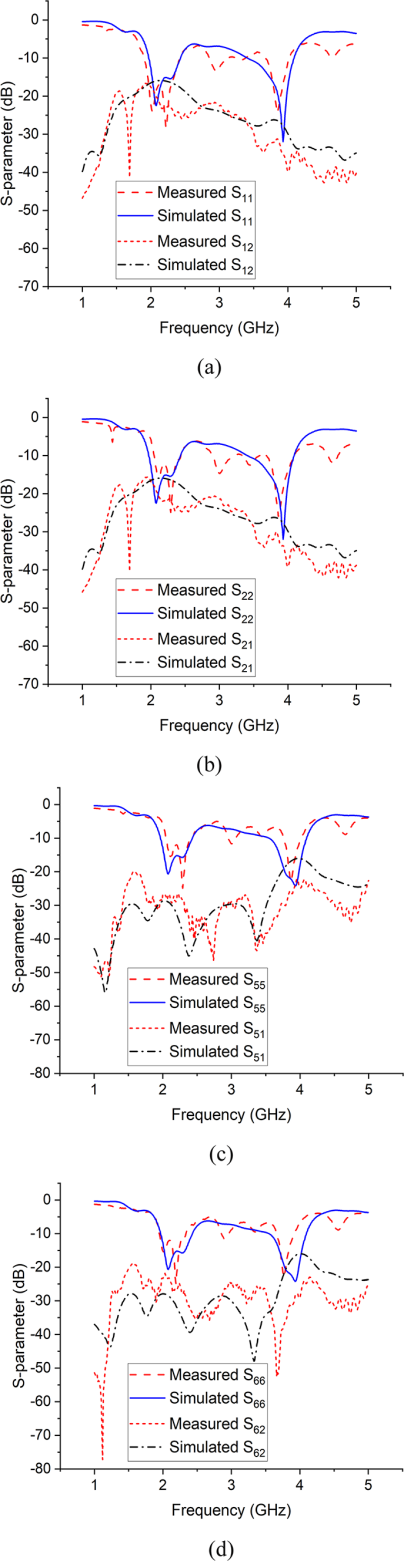
Simulated and measured S-parameters of antenna array (**a**) S_11_ and S_12_, (**b**) S_22_ and S_21_, (**c**) S_55_ and S_51_, (**d**) S_66_ and S_62_.

**Fig. 8:**
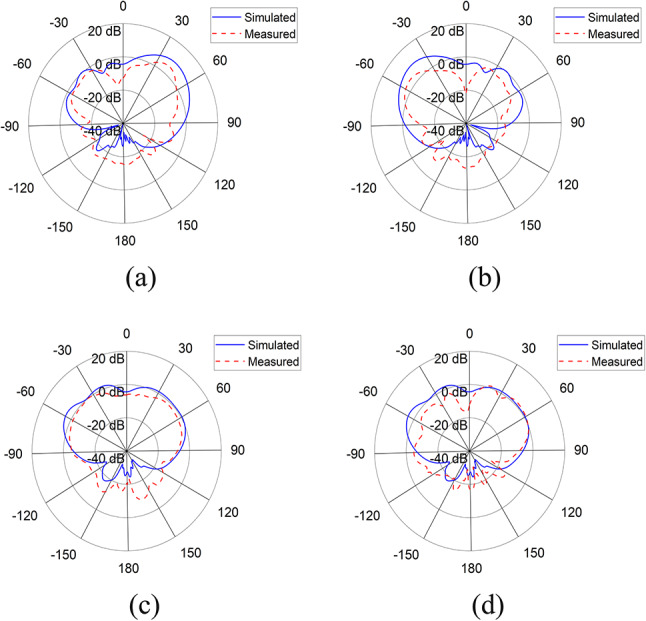
Simulated and measured radiation patterns of antenna array at 3.9 GHz in (**a**) Azimuth plane for $\:P1$, (**b**) Azimuth plane for $\:P4$, (**c**) Elevation plane for $\:P1$, (**d**) Elevation plane for $\:P4$.

The S-parameter measurements were taken using a Vector Network Analyzer and radiation patterns were measured in the Anechoic Chamber. Figure 7 shows the simulated and measured S-parameters for port 1, 2, 5 and 6. Figure 8 shows the simulated and measured radiation patterns in azimuth (xz) plane and elevation (yz) plane for port 1 and 4 of the antenna array at 3.9 GHz.

### Simultaneous excitations

The proposed array consists of eight excitation ports, with each port corresponding to an individual dipole element. This configuration allows for independent control of each dipole’s radiation pattern and characteristics. In order to increase the antenna gain, two different excitation schemes, referred to as scheme-1 and scheme-2, are employed.

**Fig. 9:**
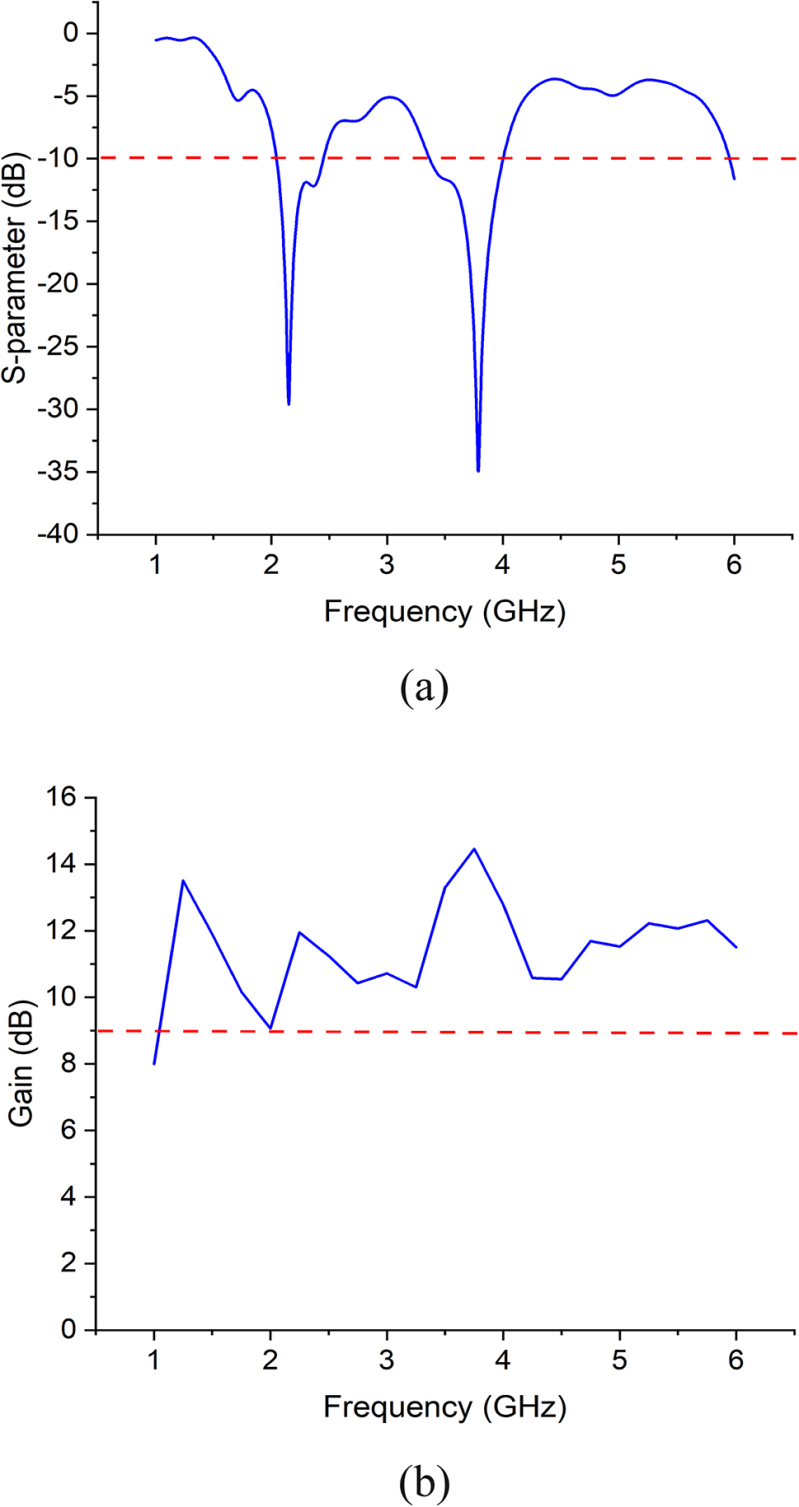
Simultaneous excitation of Scheme-1 (**a**) S-parameter, (**b**) Broadband Gain.

**Fig. 10:**
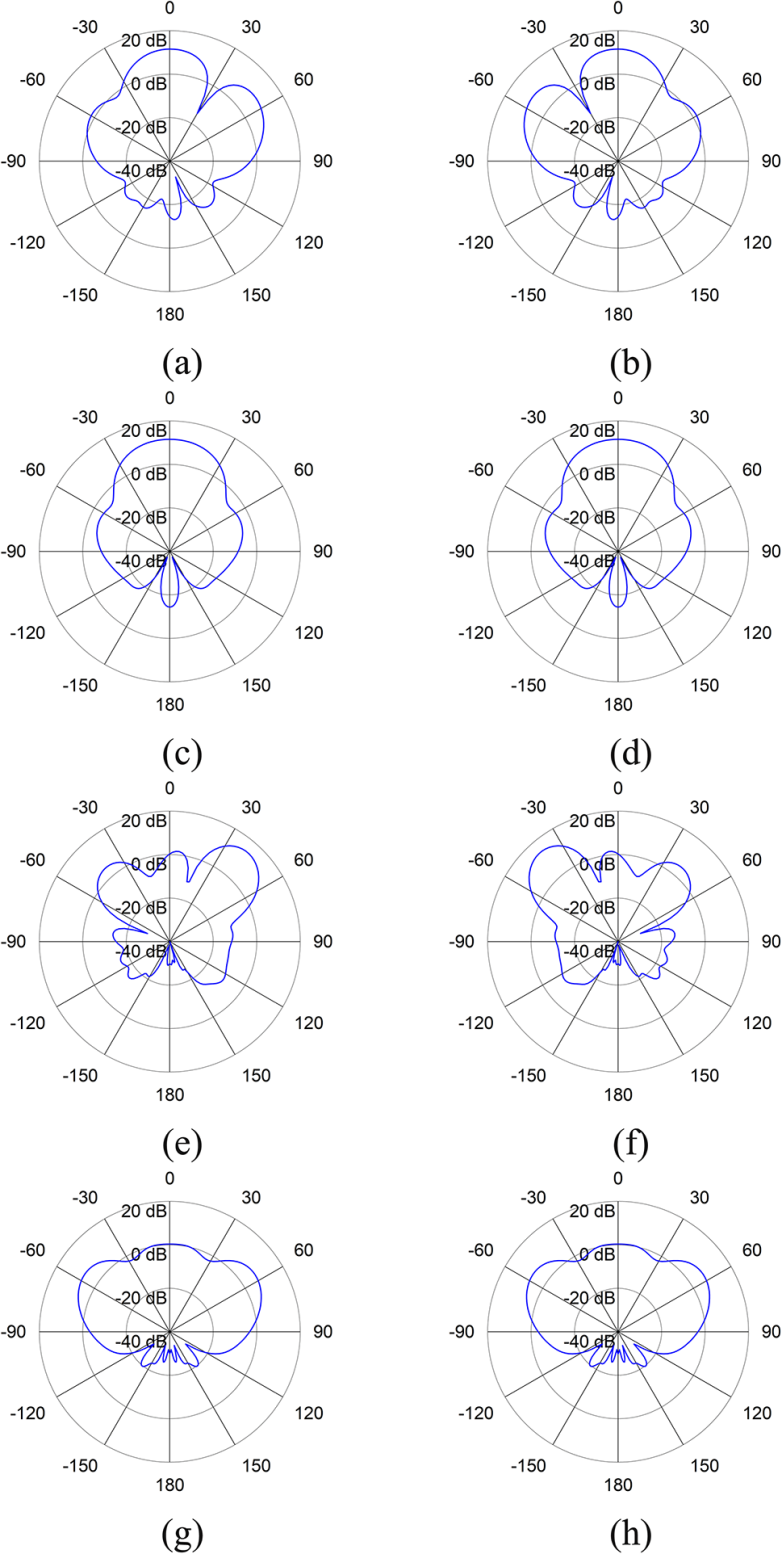
Simulated radiation patterns of antenna array with simultaneous excitation in (**a**) Azimuth plane for Scheme-1 at 2.2 GHz, (**b**) Azimuth plane for Scheme-2 at 2.2 GHz, (**c**) Elevation plane for Scheme-1 at 2.2 GHz, (**d**) Elevation plane for Scheme-2 at 2.2 GHz, (**e**) Azimuth plane for Scheme-1 at 3.8 GHz, (**f**) Azimuth plane for Scheme-2 at 3.8 GHz, (**g**) Elevation plane for Scheme-1 at 3.8 GHz, (**h**) Elevation plane for Scheme-2 at 3.8 GHz.

In scheme-1, ports 1, 3, 5, and 7, while in scheme-2, ports 2, 4, 6, and 8 are simultaneously excited. Figure 9 shows the results for simulated S-parameter and broadband gain, for scheme-1, from which it is clear that the array is resonating at 2.2 GHz and 3.8 GHz. The broadband gain indicates that the array is acting as an overall high-gain antenna system, with an overall gain higher than 10 dB in both the operating bands. The same results have also been obtained for scheme-2. Figure 10 shows the simulated radiation patterns in azimuth (xz) plane and elevation (yz) plane of the antenna array for scheme-1 and scheme-2 at 2.2 GHz and 3.8 GHz. This method of simultaneous excitation, results in a more focused antenna beam in a particular direction and thus increasing the antenna gain up to 11.5 dB at 2.2 GHz and 14.5 dB at 3.8 GHz.

From Fig. 10, it can also be seen that the proposed array provides a distinctive feature of dual sector coverage from a single antenna array panel at 3.8 GHz, as two distinct beams directed in different directions are being obtained by using scheme-1 and scheme-2. This type of technique leads to an increased coverage capacity for the 5G base station, without the use of additional system resources.

### Beam steering

In order to increase the coverage capacity of the antenna array, beam steering is proposed by using the method of phase shifting. The phase shift introduced to each signal determines the direction of the resultant radiation pattern.

**Fig. 11:**
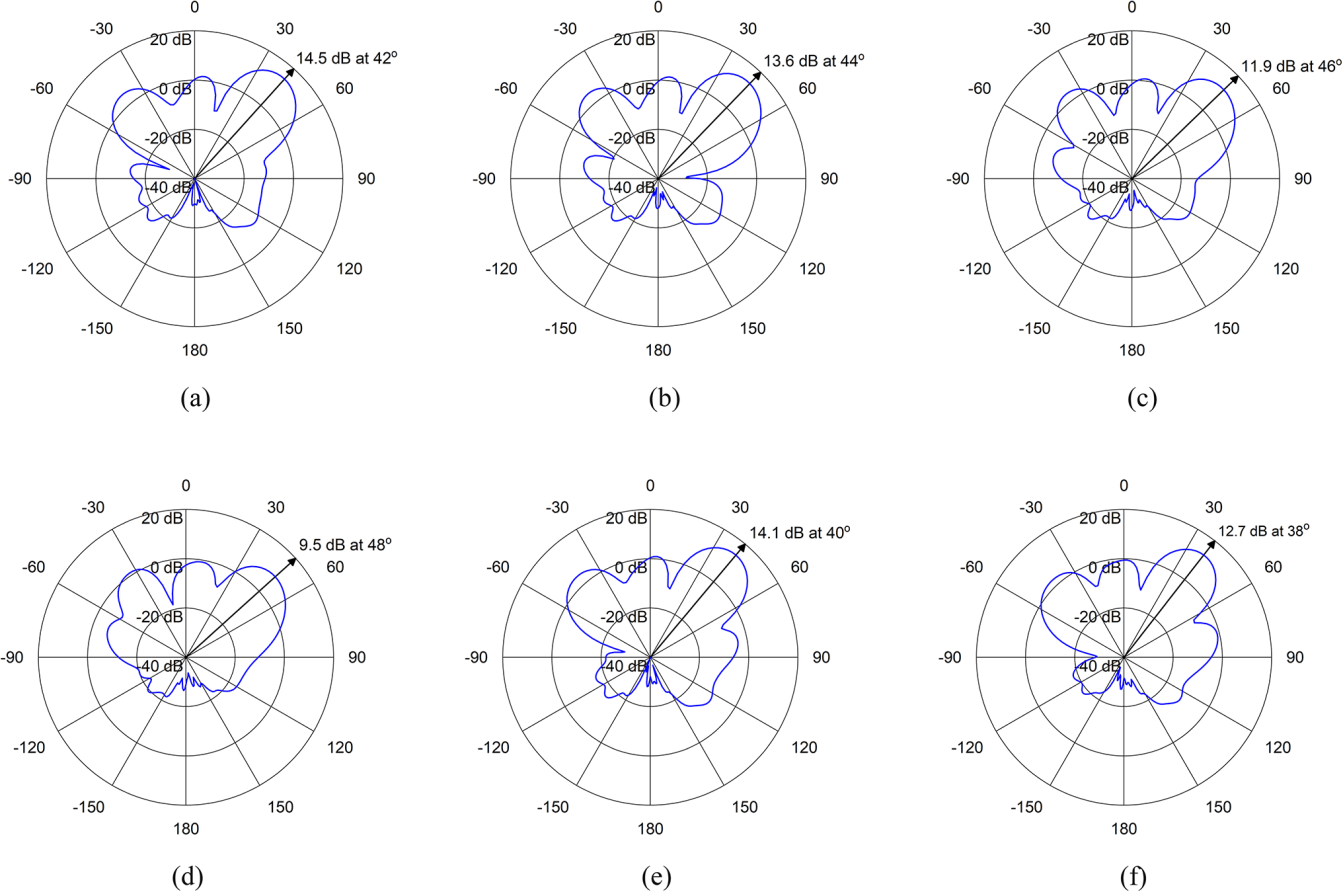
Radiation patterns for Excitation Scheme-1 (**a**) Combination-1, (**b**) Combination-2, (**c**) Combination-3, (**d**) Combination-4, (**e**) Combination-5, (**f**) Combination-6.

**Fig. 12:**
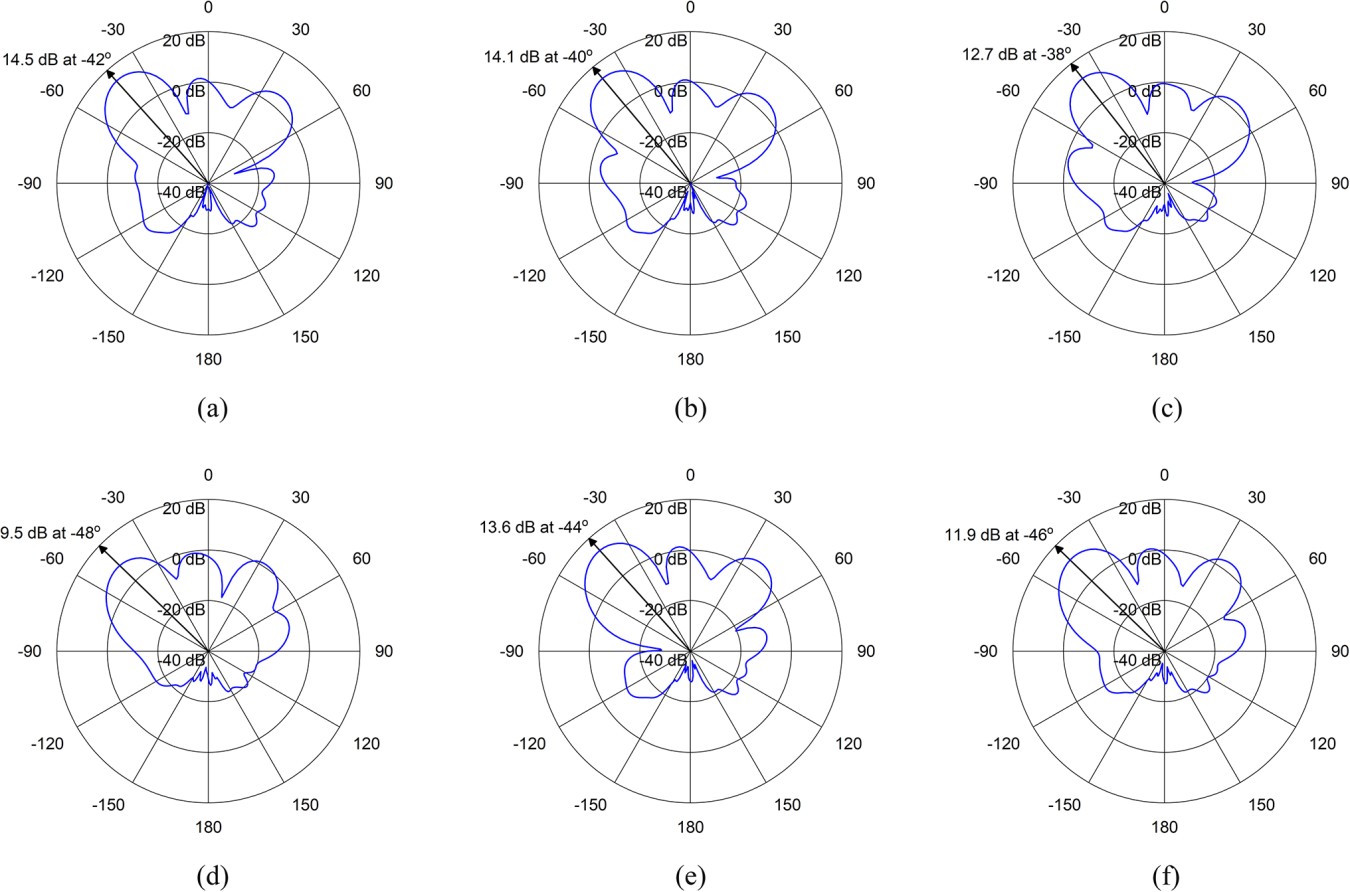
Radiation patterns for Excitation Scheme-2 (**a**) Combination-1, (**b**) Combination-2, (**c**) Combination-3, (**d**) Combination-4, (**e**) Combination-5, (**f**) Combination-6.

By adjusting the phase shifts appropriately, constructive interference can be achieved in the desired direction, while destructive interference occurs in other directions. When the phase shifts are set such that the signals from each element combine coherently in the desired direction, the array focuses its radiation pattern towards that direction. Table 3 shows the different phase combinations of the excitation signals for both the schemes at 3.8 GHz. In each scheme, the ports are applied with the excitation signals of the same magnitude but with different phase angles. The radiation patterns in azimuth (xz) plane at 3.8 GHz, for different combinations of excitation signals are shown in Figs. 11 and 12. In both the cases, the main beam is steering up to 10$\:^\circ\:$ whereas, it’s half-power beamwidth (HPBW) is varying up to 5.2$\:^\circ\:$. Therefore, the proposed array is not only working as beam steering but also as pattern reconfigurable antenna array.

## Results and discussion

A comparison between our proposed model and other high-gain 5G sub-6 GHz base station arrays is presented in Table 4, from which it is clear that our design is not only compact in terms of size and number of ports, but is also fulfilling other requirements for 5G base station transmission like dual-band operation with high-gain and beam steering capability. None of the other studies have satisfied all these conditions in a single design.

One of the advantages of using arrays is gain enhancement. The larger the array, the higher is the antenna gain. In general, we can say that by increasing the number of radiating elements i.e. the number of excitation ports, the antenna gain increases. In our design, we have achieved a higher gain by using lesser number of excitation ports as compared to other proposed designs.

In order to validate our model, both the simulated and measured results have been presented, which are mostly in agreement with each other. Figure 3 shows the antenna’s reflection and transmission coefficients. The S-parameters for port 1 and 2 show that the antenna element is resonating at 2.2 and 3.9 GHz with reflection coefficient of -40 dB and − 38 dB respectively, whereas the port to port isolation is less than − 15 dB in both frequency bands. Figure 4 shows the antenna’s radiation patterns for port 1 and 2 in azimuth and elevation planes at 3.9 GHz. In azimuth plane, the main radiating lobe has a magnitude of 8.7 dB and HPBW of 55$\:^\circ\:$ and is directed at 57$\:^\circ\:$ and − 57$\:^\circ\:$ for port 1 and port 2 respectively. Figure 7 shows the array’s reflection coefficients for port 1, 2, 5 and 6. The antenna array is resonating at 2.1 and 3.9 GHz with reflection coefficient of -22 dB and − 32 dB respectively, whereas the port to port isolation in all the cases is less than − 15 dB in both frequency bands. Figure 8 shows the array’s radiation patterns for port 1 and 4 in azimuth and elevation planes at 3.9 GHz. In azimuth plane, the main radiating lobe has a magnitude of 9.2 dB and HPBW of 30$\:^\circ\:$ and is directed at 43$\:^\circ\:$ and − 43$\:^\circ\:$ for port 1 and 4 respectively.

For using our proposed array model for base station operation, simultaneous excitation of port 1, 3, 5, 7 (scheme-1) and port 2, 4, 6, 8 (scheme-2) has been used independently. The S-parameter and the broadband gain using simultaneous excitations for scheme-1 is shown in Fig. 9. The same results have also been obtained for simultaneous excitation for scheme-2. The array has two operating bands, the first one with a center frequency of 2.2 GHz and reflection coefficient of -30 dB, whereas the second one with a center frequency of 3.8 GHz and reflection coefficient of − 35 dB. The first band has a bandwidth of 400 MHz, starting from 2.05 GHz to 2.45 GHz and the second band has a bandwidth of 700 MHz, starting from 3.3 GHz to 4 GHz. This shows that our proposed model is operating as a dual-band antenna and hence makes it suitable not only for 5G but also for 3G/4G base station transmission. The result for broadband gain indicates that the array is acting as an overall high gain antenna system, with an overall gain higher than 10 dB in both the operating bands. The array is achieving a maximum gain of 14.5 dB at 3.8 GHz. The simulated radiation patterns in azimuth plane of the antenna array for scheme-1 and scheme-2 at 2.2 GHz and 3.8 GHz are presented in Fig. 10. At 2.2 GHz, the main radiating lobe has a magnitude of 11.5 dB and HPBW of 34$\:^\circ\:$ and is directed at 5$\:^\circ\:$ and − 5$\:^\circ\:$ for scheme-1 and scheme-2 respectively. At 3.8 GHz, the main radiating lobe has a magnitude of 14.5 dB and HPBW of 20.2$\:^\circ\:$ and is directed at 42$\:^\circ\:$ and − 42$\:^\circ\:$ for scheme-1 and scheme-2 respectively. These results show that the method of simultaneous excitation, has resulted in a more focused antenna beam with a gain of 11.5 dB at 2.2 GHz and 14.5 dB at 3.8 GHz.

**Table 3:** Phase combinations for excitation schemes of the proposed base station array.

Scheme	Combination	Phase (deg.)				Main Lobe		
		Port 1	Port 3	Port 5	Port 7	Direction (deg.)	Magnitude (dB)	HPBW (deg.)
Scheme-1	1	0	0	0	0	42	14.5	20.2
	2	0	15	30	45	44	13.6	21.5
	3	0	30	60	90	46	11.9	24.1
	4	0	45	90	135	48	9.5	25.4
	5	45	30	15	0	40	14.1	20.6
	6	90	60	30	0	38	12.7	22.7

Scheme	Combination	Phase (deg.)				Main Lobe		
		Port 2	Port 4	Port 6	Port 8	Direction (deg.)	Magnitude (dB)	HPBW (deg.)
Scheme-2	1	0	0	0	0	− 42	14.5	20.2
	2	0	15	30	45	− 40	14.1	20.6
	3	0	30	60	90	− 38	12.7	22.7
	4	135	90	45	0	− 48	9.5	25.4
	5	45	30	15	0	− 44	13.6	21.5
	6	90	60	30	0	− 46	11.9	24.1

**Table 4:** Comparison of high-gain 5G Sub-6 GHz base station antenna arrays.

References	Band (GHz)	Gain (dB)	No. of ports	Physical size (cm^2^)	Electrical size ($\:{\lambda\:}_{0}$^2^)	Beam steering	Multiband/Wideband*
^11^	2.1	19	16	88 $\:\times\:$ 14.3	6.2 $\:\times\:$ 1	No	Yes
^12^	3.7	16.7	48	194.4 $\:\times\:$ 8.6	24 $\:\times\:$ 1	No	No
^13^	3.6	19.5	24	44.5 $\:\times\:$ 29.6	5.3 $\:\times\:$ 3.6	Yes	No
^14^	2.3	17.4	10	120 $\:\times\:$ 14	9.2 $\:\times\:$ 1	Yes	No
^15^	2.2	14.8	10	60 $\:\times\:$ 14	4.4 $\:\times\:$ 1	No	Yes
^16^	4.5	11	8	30.4 $\:\times\:$ 7.6	4.6 $\:\times\:$ 1.1	No	Yes
^23^	3.6	13.8	4	74.2 $\:\times\:$ 19	8.9 $\:\times\:$ 2.3	No	Yes
^25^	2.0	15.8	20	137 $\:\times\:$ 14	9.1 $\:\times\:$ 0.9	No	Yes
^37^	5.8	18	64	256 $\:\times\:$ 21.5	49.5 $\:\times\:$ 4.1	No	No
^38^	3.6	16.6	16	96 $\:\times\:$ 12	11.5 $\:\times\:$ 1.4	No	Yes
Proposed Design	3.8	14.5	8	45 $\:\times\:$ 40	5.7 $\:\times\:$ 5	Yes	Yes

*Multiband antennas operate over more than one frequency band in sub-6 GHz range whereas, wideband antennas operate in almost the entire sub-6 GHz range with the same operational characteristics.

## Conclusion

A 5G sub-6 GHz base station antenna array consisting of printed U-shaped dipoles positioned above a metal reflector, was designed and evaluated. The array operates at 2.2 GHz and 3.8 GHz with a bandwidth of 400 MHz and 700 MHz respectively. The dimensions of the reflector and the height of the dipoles are adjusted to maximize the antenna gain up to 11.5 dB at 2.2 GHz and 14.5 dB at 3.8 GHz. Beam steering up to 20$\:^\circ\:$ is accomplished by simultaneously exciting different ports with phase shifted signals. Apart from having a simple and compact design with fewer number of excitation ports, the array offers dual-band, high-gain, beam steering capability, making it a suitable candidate for 5G base station applications. Additionally, it provides a distinctive feature of covering two sectors from a single panel, leading to an increased coverage capacity for the base station, without requiring additional system resources.

## Data Availability

The data that support the findings of this study are available upon reasonable request from the corresponding author at the email: imran.aziz@physics.uu.se.
